# Evaluation of *Kabuli* Chickpea Genotypes for Terminal Drought Tolerance in Tropical Growing Environment

**DOI:** 10.3390/plants14050806

**Published:** 2025-03-05

**Authors:** Megha Subedi, Mani Naiker, Ryan du Preez, Dante L. Adorada, Surya Bhattarai

**Affiliations:** 1 School of Health, Medical and Applied Sciences, Central Queensland University, Rockhampton, QLD 4701, Australia; m.naiker@cqu.edu.au (M.N.); r.dupreez@cqu.edu.au (R.d.P.); s.bhattarai@cqu.edu.au (S.B.); 2 Centre for Crop Health, Institute for Life Science and the Environment, University of Southern Queensland, Toowoomba, QLD 4350, Australia; dante.adorada@usq.edu.au

**Keywords:** *kabuli*, chickpea, terminal drought, tropical adaptation

## Abstract

Terminal drought is the major constraint for chickpea production, leading to yield losses of up to 90% in tropical environments. Understanding the morphological, phenological, and physiological traits underlying drought tolerance is crucial for developing resilient chickpea genotypes. This study elucidates the drought-tolerant traits of eight *kabuli* chickpea genotypes under a controlled environment using polyvinyl chloride (PVC) lysimeters. Terminal drought was imposed after the flowering stage, and the response was assessed against non-stress (well-watered) treatment. Drought stress significantly impacted gas-exchange parameters, reducing the stomatal conductance (16–35%), chlorophyll content (10–22%), carbon assimilation rate (21–40%) and internal carbon concentration (7–14%). Principal component analysis (PCA) indicated three groups among these eight genotypes. The drought-tolerant group included two genotypes (AVTCPK#6 and AVTCPK#19) with higher water use efficiency (WUE), deep-rooted plants, longer maturity, and seed yield stability under drought stress. In contrast, the drought-susceptible group included two genotypes (AVTCPK#1 and AVTCPK#12) that were early-maturing and low-yielding with poor assimilation rates. The intermediate group included four genotypes (AVTCPK#3, AVTCPK8, AVTCPK#24, and AVTCPK#25) that exhibited medium maturity and medium yield, conferring intermediate tolerance to terminal drought. A significantly strong positive correlation was observed between seed yield and key physiological traits (stomatal conductance (gsw), leaf chlorophyll content (SPAD) and carbon assimilation rate (A_sat_)) and morphological traits (plant height, number of pods, and root biomass). Conversely, carbon discrimination (Δ^13^C) and intrinsic WUE (iWUE) showed a strong negative correlation with seed yield, supporting Δ^13^C as a surrogate for WUE and drought tolerance and a trait suitable for the selection of *kabuli* chickpea genotypes for drought resilience.

## 1. Introduction

Chickpea is a globally produced legume crop cultivated at 14.8 million hectares, with an annual production of 18.09 million tons harvested in 2022 [1]. In the context of Australia, chickpea is grown on 615,750 hectares across several agricultural regions. Central Queensland through to New South Wales, Victoria, and South Australia are major regions for chickpea production [1]. Although the northern region is a newer expansion of Australian chickpea production, it now contributes over 90% of the chickpea cropping area [2]. Despite Australia being the world’s largest exporter of chickpeas, exceeding a production of over 2 million tons in 2017, it has always been a challenge for farmers to protect the crop from abiotic constraints such as drought stress, which limits the potential yield [3].

As a cool season crop, 90% of Australian chickpeas are grown as rainfed crops [4]. Being a rainfed winter crop, securing adequate soil moisture for crop production is a major challenge. Since the northern Australian wet season occurs in summer and experiences a drier winter, the chickpea crop often faces challenges related to water deficits, mildly during the vegetative stage and severely during the reproductive stage of growth (terminal drought), and hence drought significantly impacts yield and quality. For instance, in the research reported by Regan, K., et al. [5] a large yield gap of 1.61 t/ha caused by water deficit was observed in chickpea in the northern region. Although disease and soil constraints were additional yield-limiting factors, water deficit was the overriding limiting factor reported in the reduction in potential yield [6]. This reduced chickpea yield affects profitability, market stability, and farm sustainability, making drought-resilient genotypes a priority for long-term production viability.

Growers and the industry have adopted different strategies to deal with drought stress in chickpea. Early planting to escape terminal drought, the use of early-maturing varieties, and deep sowing are some of the practices followed by chickpea growers in Australia [7]. Despite these adaptive measures, limited studies have been conducted in northern Australia, especially addressing the response of *kabuli* chickpea genotypes at different growth scales and development. Hence, to thrive in changing environmental conditions with limited soil moisture, there is a dire need for highly drought-tolerant chickpea cultivars suitable for this ecological region [8].

The response of a plant to a water-limited environment depends on the intensity and duration of stress, the growth stages, and the relative drought tolerance of the crop variety in use [9,10]. The plant’s ability to utilize available water varies with genotype [11,12]. There is a threshold level for soil water requirements for each genotype, below which the physiological process begins to decrease [13]. These threshold levels coincide with the reduction in leaf stomatal conductance and photosynthetic rate [14]. The study of the physiological behavior of each genotype therefore helps in understanding the water stress-tolerance level.

Under water stress, reduced plant production and growth are attributed to both stomatal and non-stomatal factors, such as stomatal closure, leaf senescence, impaired photosynthetic machinery, an increase in intercellular carbon dioxide, and chlorophyll degradation [15,16,17]. In terms of an initial response to water stress, Pouresmael, M., et al. [18] suggested that stomatal regulation was a fundamental trait necessary for drought tolerance, while Rahbarian, Khavari-Nejad [9] highlighted higher carbon assimilation rates in drought-tolerant genotypes as key traits for screening adaptation under drought-stress conditions.

Recent advancements in breeding for drought resistance have focused on traits such as harvest index (HI), water use efficiency (WUE), and leaf chlorophyll content [19]. Similarly, WUE is associated with carbon isotope discrimination (∆^13^C) in chickpea and used as a surrogate for WUE in many crops and breeding programs [20].

In a conventional breeding program, the plant’s response to drought stress is indirectly evaluated based on yield parameters such as the number of pods, seed size, and seed yield [21]. However, yield parameters alone are insufficient to screen drought-tolerance genotypes. Therefore, multiple traits, such as morphological, phenological, physiological, and biochemical governing yield under a drought environment are considered to be more reliable for screening genotypes for stable drought tolerance in chickpea [22,23].

Morphological traits such as root architecture, leaf morphology, and plant growth dynamics influence the plant’s ability to acquire and utilize water efficiently. Chickpea is classified among the drought-tolerant legumes due to its capacity for deeper root growth into the soil profile under water-stressed environments, allowing better uptake of soil resources [24]. This encourages researchers to recognize advantageous root traits that can improve crop production [25]. Further, root size, root surface area, and root length are traits that determine root water conductivity for water transport [25,26]. Hence, in this experiment, polyvinyl chloride (PVC) lysimeters were used so that root traits could be studied without damaging the root structure. Additionally, a controlled environment screening method was applied to ensure precise control over environmental factors, as physiological traits are highly dynamic and require precise timing of measurement. Furthermore, unlike the plants in the pot trials, plants grown in the field experience different levels of drought stress and are constantly exposed to greater variance in environmental conditions throughout the growing season. However, plant growth under a controlled environment allows for the identification of drought stress-tolerant traits more clearly, providing a strong basis to understand genetic control [27].

In the current study, the genotypic response of eight AgriVentis *kabuli* chickpea genotypes was evaluated under well-watered (fully irrigated) and water-stressed (ceased irrigation after flowering) conditions in a glasshouse environment. We assessed the phenological, morphological, physiological, and biochemical traits with the aim of providing valuable insights into the adaptative strategies of *kabuli* chickpea for production in tropical environments in northern Australia.

## 2. Results

### 2.1. Water Use by Plant

#### 2.1.1. Water Applied to the Plant

**Figure 1:**
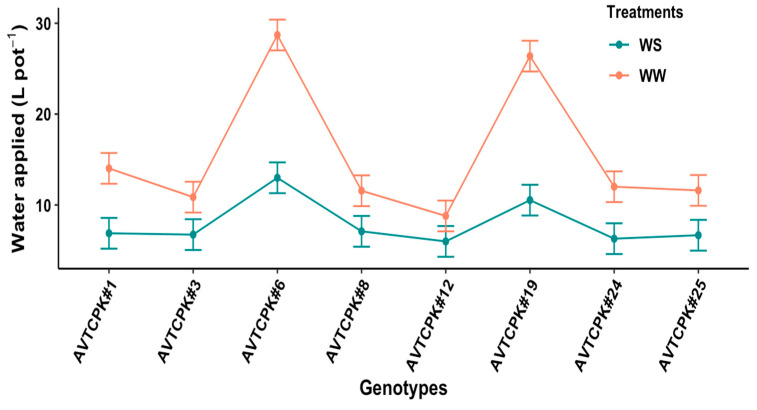
Total water applied to the plant in well-watered (WW) and water-stress (WS) treatments in eight chickpea genotypes. Each vertical bar represents the least significant difference.

The amount of water applied varied significantly among the genotypes (*p* < 0.001), treatment (*p* < 0.001), and interaction (G*T) (*p* < 0.001). Interaction between genotypes and treatment is presented in Figure 1. Cumulative water applied from sowing to maturity in the WW treatment ranged from as high as 28.7 L pot^−1^ for AVTCPK#6 to as low as 8.79 L pot^−1^ for AVTCPK#12. Similarly, in the WS treatment, water applied per plant ranged from a maximum of 13 L pot^−1^ (AVTCPK#6) to a minimum of 5.99 L pot^−1^ (AVTCPK#12). The interaction effect for applied water was due to a significant reduction in applied water for all genotypes, except for AVTCPK#12, in water-stress (WS) compared to well-watered (WW) treatment. The amount of water used per pot in the WS treatment was approximately 49% less than the WW treatment.

#### 2.1.2. Transpiration by the Plant

**Figure 2:**
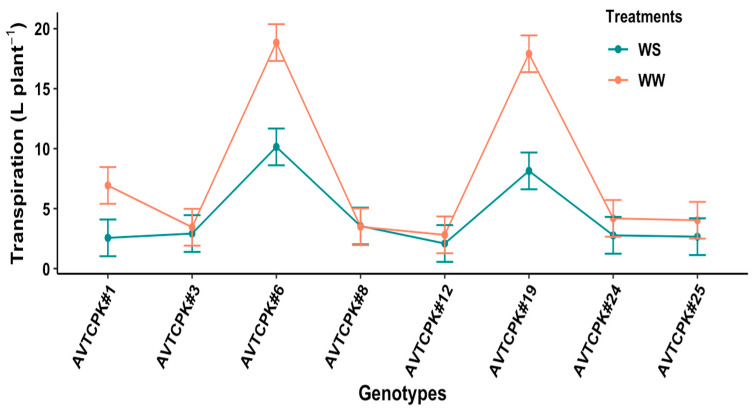
Total water transpired by the plant in well-watered (WW) and water-stress (WS) treatments in eight chickpea genotypes. Each vertical bar represents the least significant difference.

The amount of water transpired varied significantly among the genotypes (*p* < 0.001), treatment (*p* < 0.001), and interaction (G*T) (*p* < 0.001), as presented in Figure 2. Among the genotypes AVTCPK#6 had the highest water transpiration rate in both water treatments, while the lowest transpiration rate was observed in genotype AVTCPK#12. Total water transpired from sowing to harvest in WW plants was higher than from WS plants in three genotypes (AVTCPK#1, AVTCPK#6, and AVTCPK#19), while the other five genotypes had similar amounts of water transpired in both soil moisture regimes. In both soil moisture environments, AVTCPK#6 and AVTCPK#19 transpired considerably higher amounts of water compared to the other six genotypes. In contrast, genotype AVTCPK#1 transpired significantly more water than the other five genotypes in WW treatments, but was on a par with the five genotypes in the WS treatments.

#### 2.1.3. Plant Water Use Efficiency

**Figure 3:**
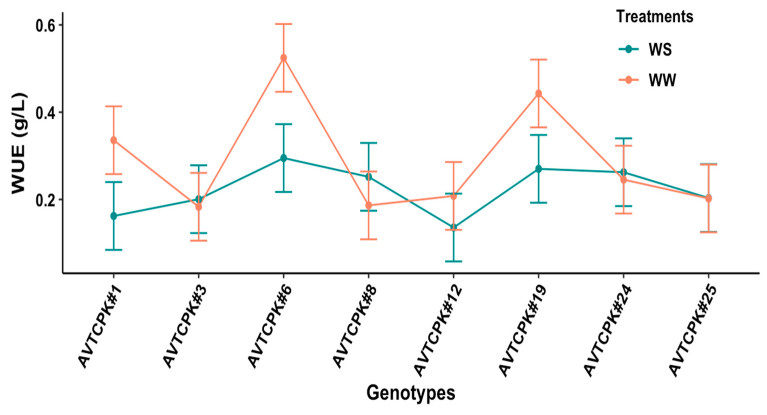
Water use efficiency in well-watered (WW) and water-stress (WS) treatments for eight chickpea genotypes. Each vertical bar represents the least significant difference.

Plant water use efficiency (WUE) differed significantly by treatment (*p* < 0.001), genotype (*p* < 0.001), and interaction (G*T) (*p* < 0.001), as presented in Figure 3. The plant WUE in general was higher for WW treatment compared to WS treatment. The WUE between genotypes did not differ significantly for WS treatment. In contrast, AVTCPK#6, AVTCPK#19, and AVTCPK#1 recorded significantly higher WUE than other genotypes in WW treatment, resulting in significant interaction effects due to genotypes x treatments (Figure 3).

### 2.2. Phenology

The time to first flowering varied significantly among the genotypes and ranged from 34 to 77 days after sowing (DAS), with late flowering in AVTCPK#6 and AVTCPK#19 (72–77 DAS) and early flowering (34–37 DAS) in other genotypes. Similarly, the days to podding also varied significantly among the genotypes, 41–85 days, with late podding in AVTCPK#6 and AVTCPK#19 (81–83 DAS) and early podding (40–45 DAS) in other genotypes.

The crop maturity not only varied with genotype but also differed significantly by irrigation treatment (Table 1). The crop maturity period ranged from 71 to 135 DAS. Two genotypes, AVTCPK#6 and AVTCPK#19, were late-maturing (112–135 DAS), whereas two genotypes, AVTCPK#1 and AVTCPK#12, were early-maturing (71–96 DAS), while the other four genotypes were medium-maturing (78–113). Crop maturing in general was delayed by WW treatment (89–135 DAS) compared to WS treatment (71–112 DAS).

### 2.3. Morphological Traits

Plant height at 60 DAS and at harvest varied significantly between genotypes and due to irrigation (Table 2). The plant height at harvest range was 33–75 cm (Table 2). The WW plants in general were taller (12%) than the WS plants, as water stress impacted on plant growth. Genotypes AVTCPK#6 and AVTCPK#19 were in general taller (67–75 cm) at harvest, whereas AVTCPK#12 and other genotypes were shorter at harvest (32–51 cm).

The number of shoots in general reduced by WS treatment at 60 DAS and at harvest (Table 3). The number of primary shoots at 30 DAS did not differ significantly among genotypes, and ranged from two to three. However, at 60 DAS, this ranged from two to five, with significant differences observed among genotypes, with AVTCPK#6 recording significantly more shoots per plant compared to other genotypes. Primary shoot counts at harvest differed significantly among genotypes and irrigation, but were not significant for the interaction (G*T).

### 2.4. Physiological Traits After Treatment Exposure

#### 2.4.1. Carbon Assimilation Rate (Asat)

**Figure 4:**
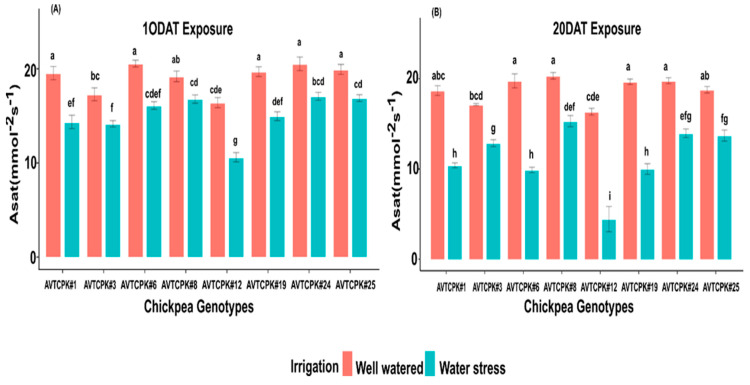
Carbon assimilation rate (Asat; mmol^−2^s^−1^) at 10 DAT (**A**) and 20 DAT (**B**) in eight chickpea genotypes. Same letters indicate, no significance, while different letters indicate a significant effect.

Carbon assimilation varied among the genotypes, treatments, and their interaction (G*T) on both measured days (Table 4 and Table 5). In WW conditions, Asat ranged from 16 to 20.5 µmol m^−2^ s^−1^. A considerable drop in Asat for WS treatment was noted on 10 DAT (21%) and on 20 DAT (40%) compared to WW plants. Severe reduction in leaf photosynthesis with prolonged WS treatment was noted for AVTCPK#12 compared to other genotypes in the trial (Figure 4).

#### 2.4.2. Stomatal Conductance Rate (gsw)

**Figure 5:**
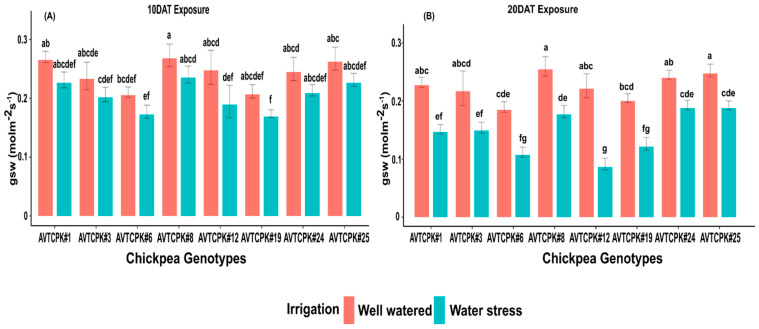
Stomatal conductance (gsw; molm^−2^s^−1^) at 10 DAT (**A**) and 20 DAT (**B**) in eight chickpea genotypes. Same letters indicate no significance, while different letters indicate a significant effect.

Stomatal conductance differed significantly among the genotypes and treatment at both stages (Table 4 and Table 5). However, a significant interaction (G*T) was also observed for 20 DAT, which is presented in Figure 5. The stomatal conductance among genotypes was 0.185–0.268 mol m^−2^ s^−1^ with the WW treatment. Higher stomatal conductance was recorded in AVTCPK#8, followed by AVTCPK#1, AVTCPK#25, AVTCPK#24, AVTCPK#12, and AVTCPK#3, while lower stomatal conductance was noted for AVTCPK#6 and AVTCPK#19 compared to other genotypes (Figure 5). A severe reduction in stomatal conductance was recorded with prolonged WS treatment for AVTCPK#12 compared to other genotypes in the trial (Figure 5).

#### 2.4.3. Internal Carbon Concentration (Ci)

**Figure 6:**
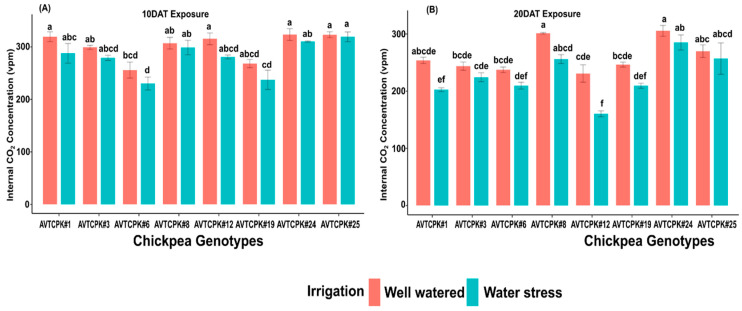
Internal CO_2_ concentration (vpm) at 10 DAT (**A**) and 20 DAT (**B**) in eight chickpea genotypes. Same letters indicate no significance, while different letters indicate a significant effect.

Significant variation was observed among the genotypes (*p* < 0.001) and treatments (*p* < 0.001), but not in interaction (G*T) for leaf internal CO_2_ concentration (Table 4 and Table 5). In the WW treatment, AVTCPK#6 and AVTCPK#19 showed lower Ci compared to other genotypes. However, in the WS condition, Ci declined in 10 DAT (6.9%) and further decreased in 20 DAT (13.6%), where genotype AVTCPK#12 showed a faster and significant decrease, unlike the AVTCPK#25 for Ci under WS treatment (Figure 6).

#### 2.4.4. Intrinsic Water Use Efficiency (iWUE)

**Figure 7:**
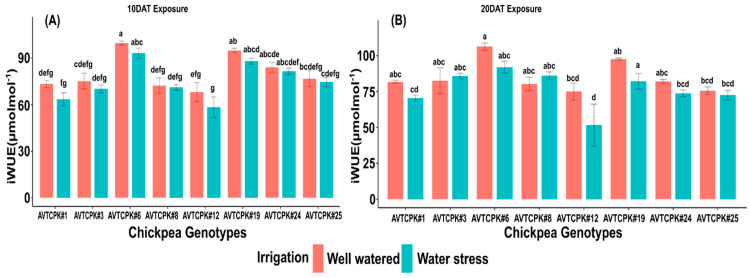
Internal water use efficiency (iWUE: (µmolmol^−1^)) at 10 DAT (**A**) and 20 DAT (**B**) in eight chickpea genotypes. Same letters indicate no significance, while different letters indicate a significant effect.

Intrinsic water uses efficiency (iWUE) varied among the genotypes (*p* < 0.001) and treatments (*p* < 0.01), but no significant was observed in the interaction (G*T) (Table 4 and Table 5). On both the measured days, iWUE was higher for AVTCPK#6 and AVTCPK#19, irrespective of irrigation treatment. Intrinsic water use efficiency decreased across all genotypes on both dates, i.e., 10 DAT and 20 DAT (Figure 7).

#### 2.4.5. Leaf Chlorophyll Content (SPAD)

**Figure 8:**
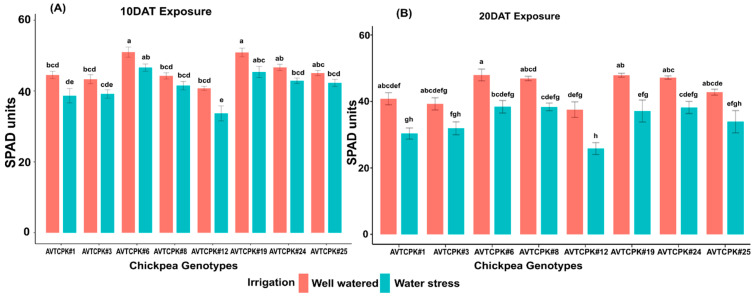
SPAD chlorophyll content (SPDA unit) at 10 DAT (**A**) and 20 DAT (**B**) in eight chickpea genotypes. Same letters indicate no significance, while different letters indicate a significant effect.

Leaf chlorophyll content, expressed as SPAD values, differed significantly between genotypes and treatments at both the measured days, but no significant interaction effect was observed for genotypes × irrigation treatments (Table 4 and Table 5). In WW treatment, SPAD chlorophyll ranged from 37 to 51. Leaf SPAD in WS treatment reduced by 10% compared to WW plants on 10 DAT. There was an increase in SPAD intensity for WS treatment at 20 DAT to 22%. The percentage reduction of chlorophyll content in genotypes AVTCPK#1 and AVTCPK#12 was comparatively higher for water-stress treatment than WW treatment (Figure 8).

#### 2.4.6. Carbon Isotope Discrimination (Δ^13^C)

Significant variation between genotypes (*p* < 0.001) and treatments (*p* < 0.001) for carbon discrimination (Δ^13^C) was recorded, but not for the genotype* treatment interaction (Table 6). Carbon isotope discrimination decreased by WS compared to WW treatment. The lowest Δ^13^C was recorded for AVTCPK#6 and AVTCPK#19, irrespective of irrigation treatments.

#### 2.4.7. Aboveground Biomass (AGB)

The aboveground biomass ranged from 4.25 to 33.6 g plant^−1^ for WW 2.4–13.2 g plant^−1^ for WS treatment, with a significant difference among genotypes (*p* < 0.001), treatments (*p* < 0.001), and interaction (G*T) (*p* < 0.001) (Table 6). The biomass yield did not reduce by WS treatment in low-yielding genotypes (AVTCPK#3, AVTCPK#8, AVTCPK#12, AVTCPK#24, and AVTCPK#25), but did reduce significantly in high-yielding genotypes (AVTCPK#6, AVTCPK#19, and AVTCPK#1).

### 2.5. Yield and Yield Attributing Traits

#### 2.5.1. Number of Pods per Plant

**Figure 9:**
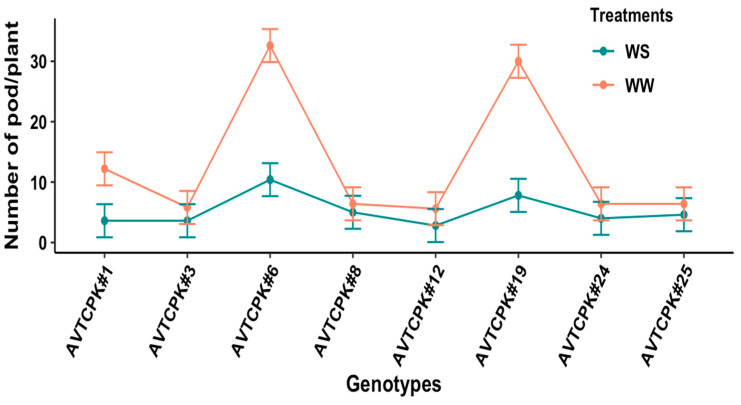
Interaction between genotype and treatment graph, presenting number of pods/plants of eight chickpea genotypes. Each vertical bar represents the least significant difference.

Significant differences in pod count were recorded for genotypes (*p* < 0.001), treatments (*p* < 0.001), and G*T interaction (*p* < 0.001). Pods per plant between genotypes ranged from 2.8 to 32.6 (Figure 9). The pod count per plant reduced significantly in WS compared to WW treatment. The pod count between WW and WS treatment did not differ significantly for low-pod-bearing genotypes (AVTCPK#3, AVTCPK#8, AVTCPK#12, AVTCPK#24, and AVTCPK#25), but did reduce significantly in high-pod-bearing genotypes (AVTCPK#6, AVTCPK#19, and AVTCPK#1).

#### 2.5.2. Pod Weight per Plant (g)

**Figure 10:**
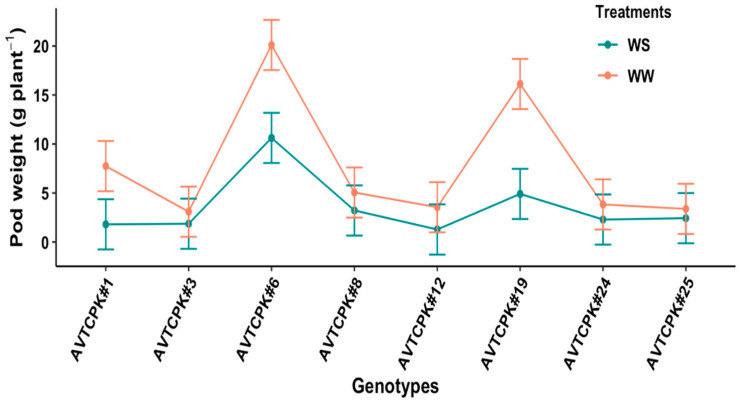
Interaction between genotype and treatment graph presenting pod weight/plant (g) of eight chickpea genotypes. Each vertical bar represents the least significant difference.

Pod weight varied significantly due to genotype (*p* < 0.001), treatment (*p* < 0.001) and interaction between genotype and treatment (*p* < 0.001). Pod weight due to WS reduced by 54.8% compared to WW treatment. The pod weight between WW and WS treatment did not differ significantly for low-pod-yielding genotypes (AVTCPK#3, AVTCPK#8, AVTCPK#12, AVTCPK#24, and AVTCPK#25), but did reduce significantly in high-pod-yielding genotypes (AVTCPK#6, AVTCPK#19 and AVTCPK#1) (Figure 10).

#### 2.5.3. Number of Seeds per Plant

**Figure 11:**
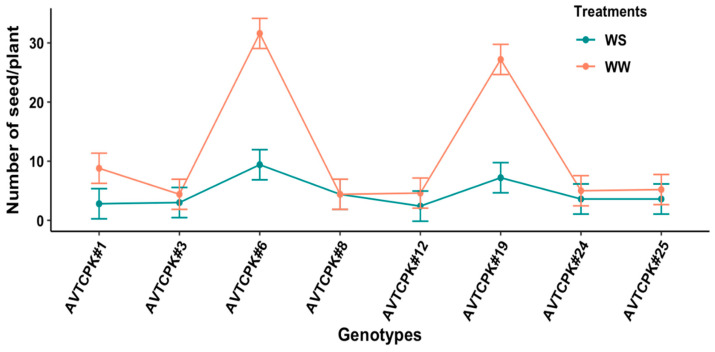
Interaction between genotypes and treatment graph presenting numbers of seeds/plants in eight chickpea genotypes. Each vertical bar represents the least significant difference.

The seed count per plant differed significantly due to genotype (*p* < 0.001), irrigation treatment (*p* < 0.001), and G*T interaction (*p* < 0.001). The WS treatment decreased the number of seeds per plant by 60% compared to WW treatment. The seed count per plant between WW and WS treatment did not differ significantly for low-seed-bearing genotypes (AVTCPK#3, AVTCPK#8, AVTCPK#12, AVTCPK#24, and AVTCPK#25), but did reduce seed count per plant significantly by WS treatment in high-seed-bearing genotypes (AVTCPK#6, AVTCPK#19, and AVTCPK#1) (Figure 11).

#### 2.5.4. Seed Yield/Plant (g)

**Figure 12:**
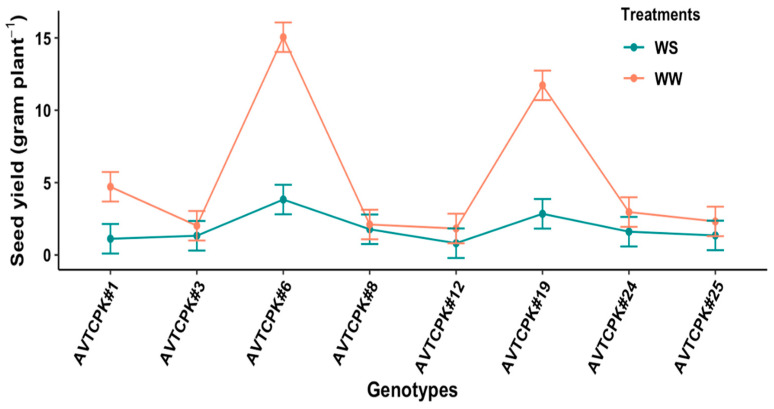
Interaction between genotypes and treatment graph presenting seed yield/plant (g) in eight chickpeas. Each vertical bar represents the least significant difference.

Seed yield of chickpea genotypes was 2.02–15.5 g/plant in WW and 0.81–3.82 g/plant in WS treatment, resulting in to yield penalty of 67% by WS treatment. A significant G*T interaction suggested that the seed yield with WS reduced significantly only for AVTCPK#1, AVTCPK#6, and AVTCPK#19, and there was no significant yield penalty with WS treatment for other genotypes (Figure 12). The seed yield between WW and WS treatments did not differ significantly for low-yielding genotypes (AVTCPK#3, AVTCPK#8, AVTCPK#12, AVTCPK#24, and AVTCPK#25), but did reduce significantly in high-yielding genotypes (AVTCPK#6, AVTCPK#19, and AVTCPK#1).

#### 2.5.5. Harvest Index

**Figure 13:**
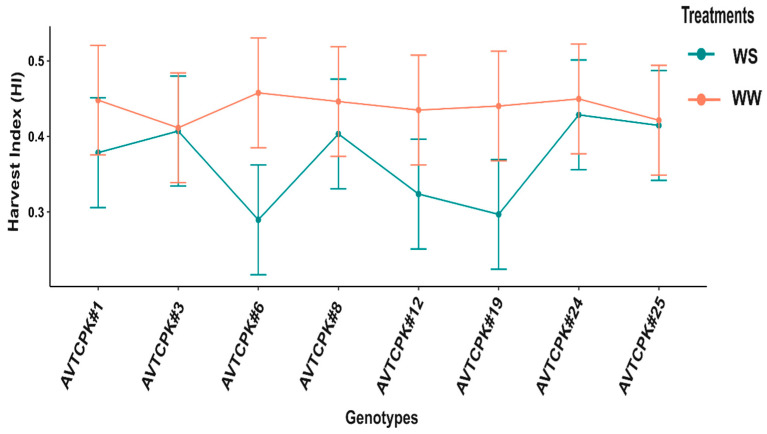
Interaction between genotypes and treatment graph presenting harvest index in eight chickpeas. Each vertical bar represents the least significant difference.

Harvest index (HI) varied significantly between treatments (*p* < 0.001) and G*T interaction (*p* < 0.01), but no significant was observed among genotypes. The HI between WW and WS treatments did not differ significantly for any genotypes, except for AVTCPK#6, where the HI reduced significantly with WS compared to WW treatment (Figure 13).

### 2.6. Root Traits

#### 2.6.1. Root Length

Irrigation treatment did not affect root length; however, variation was observed among the genotypes (Table 7). AVTCPK#6 showed the longest roots under both WW and WS, followed by AVTCPK#19, while AVTCPK#12 had the shortest roots. Other genotypes had root lengths in between.

#### 2.6.2. Root Biomass

Root biomass varied significantly among the genotypes, treatments, and G*T interaction (Table 7). Root biomass reduced significantly by WS treatment compared to WW treatment. The root biomass weight between WW and WS treatment did not differ significantly for low-biomass-yielding genotypes (AVTCPK#3, AVTCPK#8, AVTCPK#12, AVTCPK#24, and AVTCPK#25), but did reduce significantly by WS treatment in high-biomass-yielding genotypes (AVTCPK#6, AVTCPK#19, and AVTCPK#1).

#### 2.6.3. Root–Shoot Ratio

The root–shoot ratio did not differ significantly for different genotypes between well-watered and water-stress treatments, except AVTCPK#19, for which the root–shoot ratio reduced significantly for the WS treatment compared to the WE treatment (Table 7).

### 2.7. Principal Component Analysis (PCA) and Cluster Analysis

**Figure 14:**
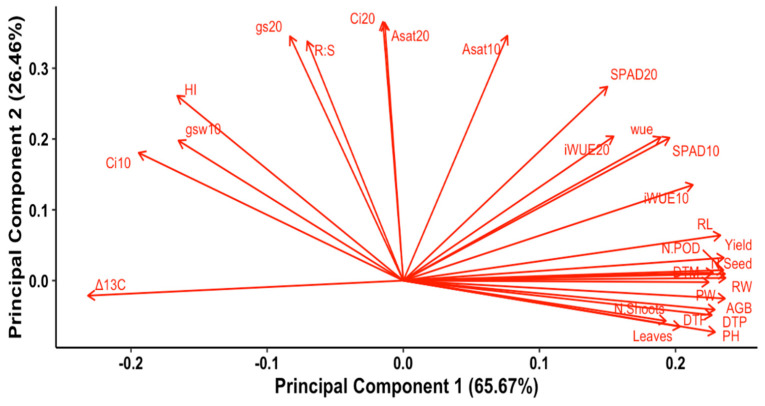
Standard PCA biplot of all traits with their loading vectors. Note: Biplot display of two principal components of all studied traits in chickpea genotypes. Correlogram showing the relationships among studied traits for water-stressed plants. SPAD10 (SPAD chlorophyll content at 10 DAT), SPAD20 (SPAD chlorophyll content at 20 DAT), Asat10 (carbon assimilation rate at 10 DAT, µmol m^−^^2^ s^−^^1^), Asat20 (carbon assimilation rate at 20 DAT, µmol m^−^^2^ s^−^^1^), gsw10 (stomata conductance at 10 DAT, mol m^−^^2^ s^−^^1^), gsw20 (stomata conductance at 20 DAT, mol m^−^^2^ s^−^^1^), Ci10 (internal carbon concentration at 10 DAT, vpm), Ci20 (internal carbon concentration at 20 DAT, vpm), iWUE10 (intrinsic water use efficiency at 10 DAT, µmol mol^−1^, iwue20 (intrinsic water use efficiency at 20 DAT, µmolmol^−1^), ∆^13^C (^13/14^carbon discrimination ratio), SY (seed yield, g plant^−1^), AGB (aboveground biomass, g plant^−1^), HI (harvest index), N.seed (number of seeds per plant), PW (pod weight, g plant^−1^), N.pod (number of pods per plant), PH (plant height at harvest, cm), PS (primary shoots at harvest), leaves (number of leaves at 60 DAS), DTF (days to flowering), DTP (days to podding), DTM (days to maturity), RL (root length, cm), RW (root dry weight, g), R:S (root–shoot ratio), WUE (water use efficiency at plant level, g/L plant) Dendrogram for eight genotypes in k-means clustering analysis is presented in Figure 16.

**Figure 15:**
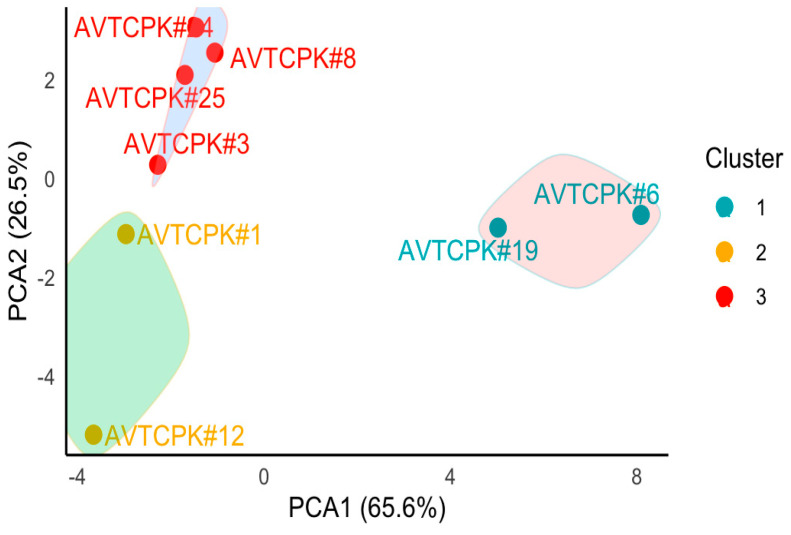
PCA scores for genotypes with grouping based on cluster analysis.

For water stress, PCA showed two components with eigenvalues greater than 1, explaining 65.64% and 26.46% of variance (Figure 14, Table 8). Growth and yield traits (ABG, root weight, seed yield (SY), pod number, plant height, root length), crop vegetative duration (days to podding (DTP), days to flowering (DTF)) correlated with drought-resistant traits. Genotypes AVTCPK#6 and AVTCPK#19 had the higher PCA values, as shown in Figure 15. ∆^13^C had a negative loading value, as opposed to PCA1. This suggests that these two genotypes with higher PCA1 values have low ∆^13^C, meaning that they use water efficiently.

On the other hand, the gas-exchange traits (Ci and A_sat_) and R:S ratio were the greatest contributors to PCA2. This suggests that if genotype selection is to be made based on these traits, AVTCPK#24 and AVTCPK#8 would be favored, as the PCA2 value was high for these genotypes, as shown in Figure 15. These genotypes exhibited strong gas-exchange activity under water stress, but since PCA2 contributed less to the variation, it played a secondary role in indicating overall drought resistance, with PCA1 being the primary.

**Figure 16:**
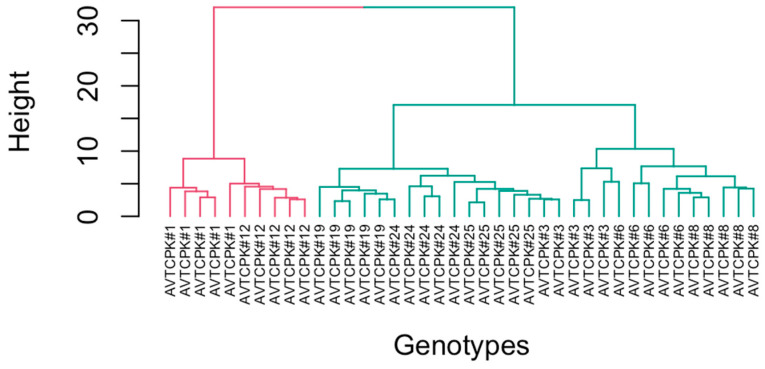
Dendrogram for eight genotypes in k-means clustering analysis.

### 2.8. Correlation Analysis

**Figure 17:**
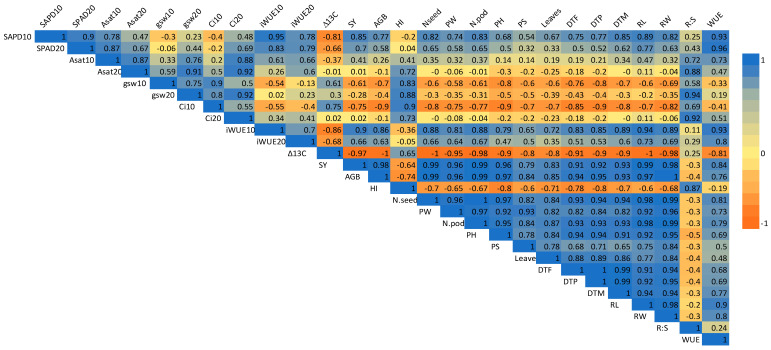
Correlogram showing the relationships between studied traits for water-stressed plants. Note: SPAD10 (SPAD chlorophyll content at 10 DAT), SPAD20 (SPAD chlorophyll content at 20 DAT), Asat10 (carbon assimilation rate at 10 DAT, µmol m^−^^2^ s^−^^1^), Asat20 (carbon assimilation rate at 20 DAT, µmol m^−^^2^ s^−^^1^), gsw10 (stomatal conductance at 10 DAT, mol m^−^^2^ s^−^^1^), gsw20 (stomata conductance at 20 DAT, mol m^−^^2^ s^−^^1^), Ci10 (internal carbon concentration at 10 DAT, vpm), Ci20 (internal carbon concentration at 20 DAT, vpm), iWUE10 (intrinsic water use efficiency at 10 DAT, µmolmol^−1^), iwue20 (intrinsic water use efficiency at 20 DAT, µmolmol^−1^), ∆13C (13/14 carbon discrimination ratio), SY (seed yield, g plant^−1^), AGB (aboveground biomass, g plant^−1^), HI (harvest index), N.seed (number of seeds per plant), PW (pod weight, g plant^−1^), N.pod (number of total pods per plant), PH (plant height at harvest, cm), PS (primary shoot at harvest), leaves (number of leaves at 60 DAS), DTF (days to flowering), DTP (days to podding), DTM (days to maturity), RL (root length, cm), RW (root dry weight, g), R:S (root–shoot ratio), WUE (water use efficiency at plant level, g/L plant).

Correlations among the studied parameters were observed for water-stressed plants (Figure 17). Among the morphology traits, plant height (r = 0.96), number of primary shoots (r = 0.79), and number of leaves (r = 0.83) showed a strong positive relationship with seed yield. Among the physiological parameters, carbon assimilation rate (A_sat_) (r = 0.41) and SPAD chlorophyll content (SPAD) (r = 0.85) were positively correlated with seed yield (SY). Similarly, strong positive correlations were observed for AGB and root length (r= 0.97) and root biomass (r= 1). Positive correlations were also noted among carbon assimilation rate (A_sat_), stomata conductance (gsw), SPAD chlorophyll content, internal carbon concentration (Ci), and intrinsic water use efficiency (iWUE). Further, iWUE showed a strong positive relationship with seed yield, while Δ^13^C showed a strong negative correlation with seed yield, hence a strong negative correlation between Δ^13^C and WUE.

## 3. Discussion

In this study, terminal drought imposed after flowering impacted a wide range of phenological, morphological, physiological, and yield-attributing traits. Consistently with previous studies on other crops, terminal drought affected a wide range of traits [13,28,29,30]. Tested genotypes were categorized into three maturity groups: early (71–96 days) (AVTCPK#1 and AVTCPK#12), medium (78–113 days) (AVTCPK#3, AVTCPK#8, AVTCPK#24 and AVTCPK#25), and late (112–135 days) (AVTCPK#6, AVTCPK#19), with specific genotypes falling into each group.

### 3.1. Effects of Water Stress on Yield and Yield-Attributing Traits

Water stress treatment reduced chickpea seed yield by 67.4% compared to the WW treatment. This reduction in yield was linked to the stress effects on pod count, seed count, and pod weight, which showed significant declines of 60.3%, 54.8%, and 61.1%, respectively. Flower and pod abortion also played a role in the reduced seed yield, as previously suggested by Pang, Turner [31] in a study on eight Desi chickpea and two commercial cultivars (Neelam and Genesis836) under terminal drought conditions that found higher (80 to 165%) flower abortion rates.

**Figure 18:**
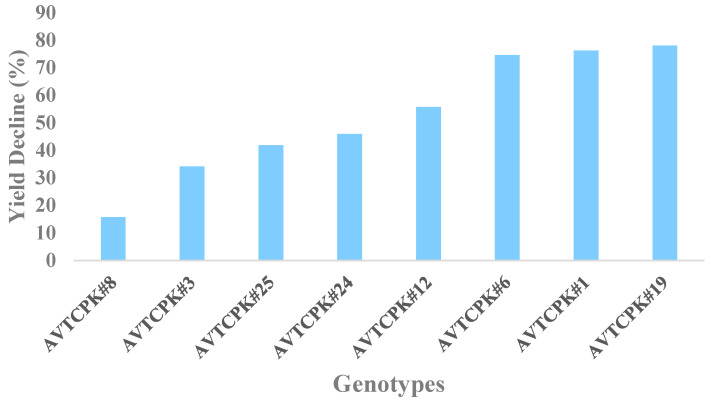
Percentage (%) decline in seed yield (g plant ^−1^) under water stress treatment relative to well-watered plant.

The decreased stomatal conductance, photoassimilation, and transpiration rate reduced assimilate supply to the growing pods, which in turn led to the reduction in seed yield, corroborating findings by Mafakheri and Siosemardeh [28]. The relative yield decline with WS treatment compared to WW treatment in the current trial is presented in Figure 18.

The early-maturing genotypes (AVTCPK#1 and AVTCPK#12) exhibited lower yield potential even in WW conditions, and their yields declined sharply under WS conditions (i.e., higher than the average >50%). Genotype AVTCPK#12 showed a lower assimilation rate, contributing to a lower seed yield in both the treatments. The medium-duration genotypes (AVTCPK#3, AVTCPK#8, AVTCPK#24, and AVTCPK#25) exhibited a medium yield decline under WS, with AVTCPK#8 showing a mere 16% decline in seed yield.

The late-maturing genotypes AVTCPK#6 and AVTCPK#19 outyielded all other genotypes in both WW and WS treatments, though they experienced a prominent yield decline under stress. The higher yielders AVTCPK#6 and AVTCPK#19 might be better able to translocate photosynthates from the leaves and stems to the early cohort of seeds that develop [13,31]. Further, these genotypes also showed higher pod numbers, pod weight, seed numbers, and aboveground biomass in both WW and WS conditions.

This result can be further linked to the biomass and root structure, where only these genotypes have compromised in biomass and maintained the higher seed yield under stress among the eight genotypes studied. Rahbarian and Khavari-Nejad [9] pointed out that plants with greater biomass indicate greater seed yield and enhanced root growth under stress conditions, which aligns with the findings of this study. Genotypes AVTCPK#6 and AVTCPK#19, with greater biomass even after reduction in WS, suggests that these genotypes could perform better in terms of biomass and seed production in field conditions owing to their deep-root traits. Genotypes AVTCPK#6 and AVTCPK#19 had comparatively profuse, longer roots, resulting in greater root dry weight, likely to extract water from deeper soil layers during water stress. Root biomass showed a similar trend to root length among the genotypes, suggesting downward elongation of roots is a major contributor to root biomass rather than lateral extension. This indicates that the increase in root length rather than lateral extension could be one potential mechanism of drought tolerance and the lysimeter height restricted the root penetration potential of the three genotypes.

The strong positive correlation observed between the seed yield and yield-attributing traits, i.e., number of seeds (0.99), pod weight (0.96), and number of pods (0.99), under water stress reinforces the importance of these traits for yield determination [32,33]. However, a negative but non-significant correlation was observed for seed yield and harvest index (HI) under water stress.

Unlike grain yield, the HI was indifferent among the genotypes in both the water regimes. However, the high-yielding genotypes AVTCPK#6 and AVTCPK#19 showed drastic drops in HI under WS compared to WW. It is evident from the results of aboveground biomass and seed yield that the two high-yielding genotypes suffered the most due to stress, exhibiting a decline in both biomass and grain yield despite higher biomass and seed yield compared to other genotypes, even under stress. However, the rate of decline in seed yield was higher compared to the biomass that resulted in such significant decline in the HI under stress. It is often reported that the HI could be one potential parameter to be considered in screening genotypes for stress tolerance [19]. But this study suggests that considering the HI as a potential indicator of stress tolerance can sometimes underestimate the production potential of the genotype and overestimate the degree of reduction in seed yield and biomass due to stress.

### 3.2. Effects of Water Stress on Physiological Traits

The literature suggests that evaluation and screening of chickpea genotypes under drought conditions on the basis of physiological parameters is a prerequisite for crop improvement [34], as it alters physiological process in plants [35].

However, a plant’s ability to inhibit reduction in photosynthesis is the crucial trait for enhancing a drought-tolerant genotype [36]. Rahbarian, Khavari-Nejad [9] studied drought-stress effects on physiological properties of four chickpea genotypes and evaluated gas-exchange parameters. Stomatal conductance (gsw), carbon assimilation rate (A_sat_), and internal carbon concentration (Ci) decreased with water stress, and this reduction was higher on sensitive genotypes (MCC68, MCC448), while tolerant genotypes showed less reduction (MCC392, MCC877). Similarly, in this study overall, reduction in gas-exchange parameters in AVTCPK#1 and AVTCPK#12, indicating even moderate water stress, was stressful for these genotypes. AVTCPK#3, AVTCPK#6, AVTCPK#8, AVTCPK#19, AVTCPK#24, and AVTCPK#25 showed a gradual decline. A positive correlation was observed between seed yield and carbon assimilation rate (A_sat_) under water stress in this study. Similarly, significant positive correlations were observed among the gas-exchange parameters.

As an initial response to drought, plant close the stomata, restricting the exchange of gas between atmosphere and leaf [37]. Restriction of CO_2_ diffusion into the leaf and also inhibition of biochemical processes such as ATP synthase and Rubisco activity results in a decreasing carbon assimilation rate and internal carbon concentration of plant [36,38]. A similar response was observed in the present study, as a significant reduction in carbon assimilation rate (A_sat_), stomata conductance (gsw), and internal carbon concentration (Ci) under water stress after the flowering stage was observed, which aligns with the studies conducted by [9,13,39] on drought and chickpeas in different locations. The non-stomatal mechanism of chlorophyll content helps to absorb light energy used for photosynthesis. Dehydration of the plant declines the light-harvesting capacity of chlorophyll, reducing chlorophyll content [40]. In our study, declines in SPAD chlorophyll content were observed in all the genotypes at WS condition.

Generally, the WUE of a plant increases with increased water stress, with tolerant genotypes delivering higher WUE than drought-sensitive genotypes [9]. In this experiment, genotype AVTCPK#6 and AVTCPK#19 maintained higher WUE at both the leaf level and plant level in both the treatments, suggesting their superior ability to cope with water stress. Other genotypes exhibited comparatively lower WUE. A significant positive relationship was observed between seed yield and WUE, as well as between chlorophyll content and seed yield, indicating that drought-tolerant genotypes could maintain higher WUE and chlorophyll levels under stress.

Leaf ∆^13^C value decreases under water stress [41], consistent with the findings from the current experiment. As a proxy measure, a decline in ∆^13^C value indicates the plant has maintained higher WUE in terms of biomass accumulation. Decline in ∆^13^C in water-stressed plants also indicates the rapid stomatal closure limiting the gas exchange [41]. As a result, the plant utilizes the available carbon inside the stomatal chamber thus, increasing the leaf ∆^13^C. In the current experiment, positive correlations were observed in ∆^13^C and internal carbon concentration, as described by Farquhar, O’Leary [42]. Negative correlations were observed between ∆^13^C and iWUE under water stress, which aligns with the finding from [41,43].

### 3.3. Effects of Water Stress on Morphological and Phenological Traits

High correlation coefficients were observed in both WW and WS treatments for seed yield and growth traits in this study, consistent with the finding from Navkiran Randhawa [33] on chickpea genotypes. Significant differences in morphological traits were observed among the eight genotypes, with genotypes AVTCPK#6 and AVTCPK#19 with maximum plant height, number of shoots, number of leaves, biomass, and root system compared to other six genotypes. Drought stress showed prominent effects on plant height and numbers of leaves and shoots of plants due to the decreased cell division and cell elongation and impaired water flow from xylem to elongating cells [33,44,45]. However, the effect of interaction (G*T) was non-significant, indicating the relative growth of genotypes was comparable across irrigation treatment.

Pappula-Reddy [39] reported a decrease in plant height by 13–25% in response to drought stress in chickpea genotypes, with the tallest plant height in the well-watered condition observed for ICC4958 (86.5 cm) and PUSA362 (772.8 cm). As observed in our study, high correlation coefficients were observed in both well-watered and water stress treatments for seed yield and growth traits. This is consistent with the results of [33].

As in this study, several other studies have reported evidence of drought-avoidance traits exhibited by chickpea genotypes under water stress [13,31]. However, in this study, it was revealed that the ability to extract available water with deeper root systems positively impacted the yield under drought, even for the late-maturing genotypes, such as AVTCPK#6 and AVTCPK#19.

## 4. Materials and Methods

### 4.1. Test Environment and Growth Conditions

The experiment was conducted in a glasshouse at CQIRP, Central Queensland University, Rockhampton (23.37° S 150.52° E), Australia in 2023 under optimum growth conditions with 23 °C and 13 °C average maximum and minimum air temperature and a mean relative humidity of 59%.

Chickpea seeds were shown in polyvinyl chloride (PVC) lysimeters 15 cm in diameter and 75 cm high (depth), closed at the base with PVC end cap to hold potting mixture. The PVC end cap consisted of four holes (each 5 mm in diameter) to ensure proper drainage. Each lysimeter was filled with 12 kg fresh weight of multipurpose potting mixture (Giru Organics, Shaw Australia) from the local Bunnings Warehouse. The moisture content in the fresh potting mixture was 52%.

### 4.2. Seed Source and Inoculation

The seeds of all eight genotypes were sourced from the Australian seed technology company AgriVentis Technologies Ltd. (Sydney, NSW, Australia) (https://www.agriventis.tech/) accessed on 2 March 2024)). The genotypes used in the experiment were AVTCPK#1, AVTCPK#3, AVTCPK#6, AVTCPK#8, AVTCPK#12, AVTCPK#19, AVTCPK#24, and AVTCPK#25. The seeds were immerged in 1% chlorine (*v*/*v*) solution for 1 h, washed thoroughly with distilled water three times, and inoculated with a peat-based slurry for chickpea (Group N, CC1192 nodulators, using Nodulaid^®^ by BASF, Southbank, Australia). Two seeds were hand-sown at a depth of 10 cm in each lysimeter on 12th March 2023. Thinning was conducted two weeks after the emergence of one seedling in each lysimeter. Fertilizer (@Yates Thrive all-purpose soluble fertilizer, Clayton, Australia) was applied as a solution of 25:5:8.8% NPK *w*/*w* at a rate of 20 mL/plant twice: in the third and fourth week after sowing. Mancozeb plus garden fungicide and miticide were prepared (5 g/L) and applied at a rate 10 mL/plant thrice at 30 DAS, 40 DAS, and 50 DAS.

### 4.3. Experimental Design and Treatments

The experiment was laid out in a completely randomized design (CRD) with two factors replicated five times. Factor 1 consisted of eight genotypes of *kabuli* chickpea, while factor 2 consisted of two levels of soil moisture. The levels of soil moisture in the experiment consisted of a well-watered (WW) treatment, where soil moisture was maintained at field capacity (FC), and a water-stress (WS) treatment, where the soil moisture was kept at FC until flowering, but no irrigation was provided after flowering to mimic the terminal drought.

**Figure 19:**
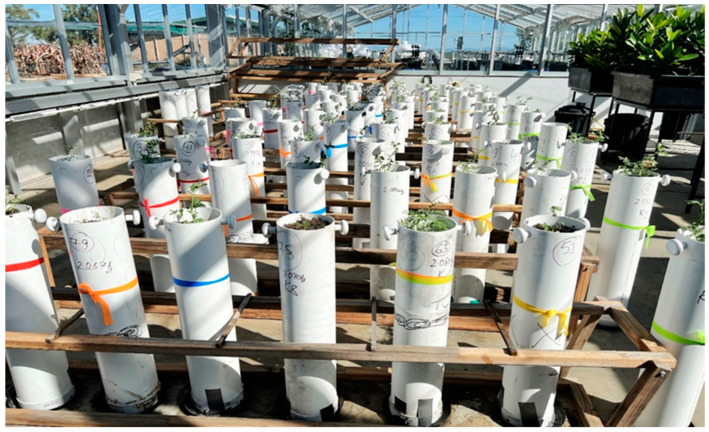
Trial setup of eight AgriVentis chickpea genotypes under well-watered (WW) and water-stressed (WS) conditions in glasshouse.

The initial moisture content, FC of potting mixture, and amount of water needed to bring the potting mixture to FC in lysimeter were determined before starting the trial following the protocol by Imakumbill [46]. The initial water content and FC were measured by gravimetry as an average taken from two lysimeter samples, each with 12 kg of potting mixture. Field capacity was determined three days later after the sample lysimeters were allowed to sit after saturation. All the lysimeters were weighed before and after each irrigation to determine irrigation requirements and the volume of water use/loss from the lysimeter. In the WS treatment, irrigation was ceased after the plants had at least one fully open flower. Out of 80 lysimeters, 40 lysimeters had irrigation ceased, while the remaining 40 lysimeters continued to receive full irrigation until the end of trial. An additional four lysimeters without plants were used, two for each treatment to estimate evaporative losses in the same environment. The setup of the lysimeter and plant-growing conditions is presented in Figure 19.

### 4.4. Data Collection

#### 4.4.1. Plant Water Use

- All lysimeters were weighed manually each week throughout the trial to assess the amount of water lost through plant transpiration (T) use and evaporation (E).
- Water use efficiency: The crop water use efficiency (WUEg) of grain production was determined as the ratio of grain yield/total water applied per lysimeter. Transpiration (T) of each plant was also determined by subtracting evaporative loss (Es), recorded as the water lost in the lysimeters without plants from the consumptive use (ET) recorded in each lysimeter with plants with corresponding water treatment, i.e., T = ET − Es [41].

#### 4.4.2. Plant Phenology and Growth Attributes

The plant phenological parameters were recorded as days to flowering (DTF), podding (DTP), and maturity (DTM) in each treatment. The growth attributes recorded in the experiment were plant height, number of leaves, and number of primary shoots, which were recorded at 30 days after sowing (DAS), 60 DAS, and at the time of harvest.

#### 4.4.3. Physiological Parameters

The following physiological parameters were assessed.

- Leaf gas-exchange parameters: The gas-exchange data were recorded using an open gas-exchange system of an infrared gas analyzer (IRGA) with an integrated fluorometer (Li-6800 Multiphase FlashTM Fluorometer, Portable Photosynthesis System, LiCor, Lincoln, NE, USA) with a leaf surface area of 1 cm^2^ and an ambient CO_2_ concentration of 370 µmol m^−^^2^ s^−^^1^ at 10 and 20 days after treatment (DAT) initiation. The parameters studied were stomatal conductance (gsw), internal CO_2_ concentration in the leaf (C_i_), and carbon assimilation rate (A_sat_). Further, intrinsic water use efficiency (iWUE) was determined as the ratio of carbon assimilation rate (A_sat_) to stomatal conductance (gsw).
- Chlorophyll content: SPAD chlorophyll content was recorded on two occasions, 10 and 20 DAT (same day as the gas-exchange parameters), from a fully expanded topmost leaf. Measurements were obtained using a Konica Minolta SPAD 502 m (Osaka, Japan).
- Carbon discrimination (Δ^13^C): The carbon discrimination (∆^13^C) ratio was measured from a fully expanded uppermost leaf that was hand-clipped at the end of reproductive stage. The leaf samples were then oven-dried at 60 °C for 48 h and ground into a fine powder using a 1 mm sieve. Approximately 6–7 mg of the resultant powder was loaded into tin capsules and placed in a 96-well sample tray. The samples were analyzed at the stable isotope laboratory (SIL) at Griffith University, Australia for C isotopes using isotope ratio mass spectrometry (CF-EA-IRMS) in an EA 1108 CHN elemental analyzer (Thermo Fisher, Milan, Italy) coupled with a Delta Plus mass spectrometer (Thermo Fisher, Milan, Italy). The result received was the ratio of ^13^C to ^12^C, usually expressed as δ^13^C_leaf_. The final discrimination for the ^13^C isotope (Δ) by the plants, compared to the atmosphere carbon isotope, was determined using the formula by Kohn [47]. The V-PBD value of air (δ^13^C_air_) was assumed to be −8% [48].

Δ^13^C = (δ^13^C_air_ − δ^13^C_leaf_)_/_(1 + δ^13^C_leaf_)

#### 4.4.4. Harvesting and Yield Assessment

Plants were harvested at 90–95% pod maturity by cutting the plants from the base (at the surface of the potting mix). Pods were detached, and number of pods, pod weight, number of seeds, and seed yield (g) was recorded for each plant. All the aboveground biomass except the seeds was oven-dried at 65 °C for 72 h for dry weight determination. Harvested seeds were air-dried, and seed yield per plant was recorded. By weighing the aboveground biomass (g DW plant ^−1^), the harvest index (HI) was determined.

#### 4.4.5. Roots and Root Traits

Roots were taken out from the lysimeter carefully from the potting mixture, rinsed with tap water, and the length noted. The roots were oven-dried at 65 °C for 72 h to record the weight of belowground biomass (g DW plant ^−1^). The root–shoot ratio was calculated as the ratio of belowground biomass and aboveground biomass (AGB).

### 4.5. Data Analysis

All the collected data were analyzed using two-way analysis of variance (ANOVA) in R version 4.2.1. The F ratios were used to note treatment significance (*p* < 0.05), and mean separation was carried out using the least significant difference (LSD). Correlations between the observed variables were determined using Pearson’s method. Principal component analysis (PCA) was performed in R version 4.2.1 using the recorded parameters. PCA is a method that reduces the complexity of the data set to a lower dimension, but retains a majority of the variation in the data set [49]. The first principal component captures the maximum variance in the data and shows the direction of highest variability in the data. The higher the variability observed in the first component, the richer the information shared by the component. PCA1 has the largest variability among all the components [50]. There were eight genotypes in this PCA study, and different traits were included.

## 5. Conclusions

Terminal drought significantly impacts the yield penalty in *kabuli* chickpeas, affecting the growth, yield attributes, and gas-exchange characteristics across different genotypes. The genotypes evaluated in this study displayed varied levels of drought tolerance, with three different maturity groups identified based on the principal component analysis (PCA).

The late-maturing genotypes, AVTCPK#6 and AVTCPK#19, outyielded under well-irrigated treatment and maintained higher yields with water-stress treatment. In these two genotypes, the drought tolerance was largely conferred by deeper and larger root systems underpinning the morphological basis and greater stomatal control and lower carbon discrimination underpinning the physiological basis.

Early-maturing genotypes (AVTCPK#1 and AVTCPK#12) showed low yield potentials under well-irrigated and water-stress conditions and a lack of significant yield difference between irrigation treatments. Owing to their short plant structure and early maturity, they are less prolific in their growth and yield features, producing the least biomass and seed yield both with and without stress. This clarifies that the escape mechanism is not sufficient to overcome yield losses by terminal drought environment and may require prolonged drought treatment for fully operational physiological and morphological tolerance mechanisms to take effect. In contrast, the medium-maturity group (AVTCPK#3, AVTCPK#8, AVTCPK#24 and AVTCPK#25) exhibited medium growth duration, medium yield, and moderate tolerance to stress. Overall, AVTCPK#6 and AVTCPK#19, with higher yielding capacity, higher WUE at both leaf and plant level, and larger root systems, are elite genotypes among the eight for drought tolerance, and are thus recommended for evaluation in field trials to assess their drought tolerance under a targeted production environment.

This research underpins how root traits, photosynthetic efficiency, and water use efficiency (WUE) are essential in enhancing drought tolerance and seed yield in chickpea. The late-maturing varieties AVTCPK#6 and AVTCPK#19, with deep root systems, higher WUE, and improved photosynthetic capacity, gave higher yields. This makes them preferable candidates in breeding for drought resistance and for plantations in areas with irregular rainfall patterns.

Furthermore, this study also highlighted a significant decline in the HI under water stress in high-yielding genotypes and points to why multiple physiological, morphological and biochemical traits should be considered for reliable selecting of drought-resistant genotypes. Similarly, traits such as root architecture, stomatal regulations, and carbon assimilation efficiency need to be considered. Breeding programs should focus on increasing traits such as root depth and photosynthetic efficiency, and selecting these traits will make it possible to develop a variety that can overcome water stress and deliver high and consistent yields.

More research should be undertaken to explore how drought stress and temperature extremes interact and how these varieties with deep root systems respond to the combined stress. This research will help find varieties that can respond to both high temperature and water scarcity, conditions that are expected to become more frequent with climate change and global warming. In addition, molecular studies focused on genes could aid in genetic modification or marker-assisted selection of resistant varieties.

## Figures and Tables

**Table 1:** Chickpea crop duration days for flowering, podding, and maturity for eight genotypes under well-watered and water-stress treatments.

Genotypes	Day to Flowering (DTF)			Day to Podding (DTP)			Day to Maturity (DTM)		
	WW	WS		WW	WS		WW	WS	
AVTCPK#1	35 ^c^	35.2 ^c^		41.6 ^c^	42.0 ^bc^		96.8 ^ef^	77.2 ^hi^	
AVTCPK#3	35.2 ^c^	36 ^bc^		43.8 ^bc^	42.6 ^bc^		101.2 ^de^	78.2 ^hi^	
AVTCPK#6	73.6 ^a^	72.6 ^a^		82 ^a^	81.2 ^a^		125.6 ^ab^	111.8 ^cd^	
AVTCPK#8	37.6 ^bc^	36.6 ^bc^		41.4 ^c^	41.6 ^c^		111.6 ^cd^	81.2 ^ghi^	
AVTCPK#12	35.6 ^bc^	34.2 ^c^		41.2 ^c^	39.4 ^c^		89.6 ^fg^	71.0 ^i^	
AVTCPK#19	76.6 ^a^	76.2 ^a^		85 ^a^	82.4 ^a^		134.8 ^a^	116.0 ^bc^	
AVTCPK#24	35.2 ^c^	36.8 ^bc^		41.2 ^c^	44.4 ^bc^		113.2 ^c^	81.8 ^gh^	
AVTCPK#25	36.8 ^bc^	38.6 ^b^		43.2 ^bc^	47.4 ^b^		111.4 ^cd^	85.0 ^ghi^	
Mean	45.70	45.75		85	82.4		111.6	87.8	
	F-value	*p*-value	LSD	F-value	*p*-value	LSD	F-value	*p*-value	LSD
Genotype (G)	510.18	<2 × 10^−16^	2.24	353.89	<2 × 10^−16^	2.80	31.31	<2 × 10^−16^	7.81
Treatment (T)	0.45	0.51	1.12	0.08	0.78	1.14	135.48	<2 × 10^−16^	3.90
G*T	1.76	0.11	3.16	1.44	0.20	3.96	1.26	0.28	11.04

Same letters indicate no significance, while different letters indicate a significant effect. LSD indicates least significant difference. Values are presented as averages ± SEM.

**Table 2:** Chickpea plant height at different growth stages and height of first pod at harvest.

Genotypes	Plant Height 30 DAS (cm)			Plant Height 60 DAS (cm)			Plant Height at Harvest (cm)		
	WW	WS		WW	WS		WW	WS	
AVTCPK#1	33.1 ± 3.2 ^a^	27.9 ± 1.8 ^ab^		44.4 ± 2.9 ^a^	32.8 ± 0.7 ^ab^		51 ± 2.5 ^cd^	35.4 ± 1.5 ^e^	
AVTCPK#3	27.9 ± 1.8 ^ab^	25.3 ± 3.2 ^ab^		37 ± 1.4 ^ab^	31.2 ± 2.7 ^ab^		42.2 ± 1.5 ^de^	32.8 ± 1.9 ^e^	
AVTCPK#6	22.2 ± 1.0 ^ab^	22.3 ± 1.8 ^ab^		41.4 ± 3.1 ^ab^	40.4 ± 1.9 ^ab^		75 ± 2.6 ^a^	66.6 ± 3.6 ^ab^	
AVTCPK#8	31.5 ± 3.1 ^a^	32.3 ± 3.1 ^a^		35.4 ± 2.5 ^ab^	37.8 ± 3.4 ^ab^		41.2 ± 1.2 ^de^	41.2 ± 1.9 ^de^	
AVTCPK#12	33.2 ± 2.4 ^a^	34 ± 1.9 ^a^		39 ± 3.9 ^ab^	36.6 ± 2.1 ^ab^		40.4 ± 2.9 ^de^	39.8 ± 3.8 ^de^	
AVTCPK#19	18.5 ± 2.1 ^b^	16.6 ± 1.9 ^b^		30.4 ± 2.9 ^b^	30 ± 3.9 ^b^		60.4 ± 6.2 ^bc^	58.4 ± 1.7 ^bc^	
AVTCPK#24	33 ± 2.1 ^a^	27.2 ± 3.3 ^ab^		38.8 ± 3.4 ^ab^	33.6 ± 1.7 ^ab^		43.2 ± 3.3 ^de^	38 ± 2.0 ^de^	
AVTCPK#25	24.9 ± 2.0 ^ab^	25.1 ± 1.6 ^ab^		30.6 ± 1.4 ^ab^	32.4 ± 2.5 ^ab^		41 ± 2.6 ^de^	36.4 ± 1.4 ^e^	
Mean	28.02	26.37		37.13	34.35		49.21	43.45	
	F-value	*p*-value	LSD	F-value	*p*-value	LSD	F-value	*p*-value	LSD
Genotype (G)	10.53	9.96 × 10^−9^	4.712	3.594	0.0025	5.39	34.84	<2 × 10^−16^	5.69
Treatment (T)	2.08	0.154	2.35	4.238	0.044	2.69	16.68	<2 × 10^−16^	2.89
G*T	0.63	0.728	6.67	1.47	0.194	7.62	1.62	0.145	8.06

Same letters indicate no significance, while different letters indicate a significant effect. LSD indicates least significant difference. Values are presented as averages ± SEM.

**Table 3:** Chickpea number of primary shoots at different growth stages.

Genotypes	Primary Shoot 30 DAS			Primary Shoot 60 DAS (cm)			Primary Shoot at Harvest		
	WW	WS		WW	WS		WW	WS	
AVTCPK#1	3.2 ± 0.2 ^a^	2.2 ± 0.5 ^a^		3.2 ± 0.2 ^abc^	2.2 ± 0.6 ^bc^		3.2 ± 0.2 ^ab^	2.2 ± 0.5 ^b^	
AVTCPK#3	2.6 ± 0.3 ^a^	2 ± 0.5 ^a^		3 ± 0 ^abc^	2.4 ± 0.4 ^bc^		3 ± 0 ^ab^	2.6 ± 0.5 ^ab^	
AVTCPK#6	3.2 ± 0.2 ^a^	2.8 ± 0.2 ^a^		4.8 ± 0.6 ^a^	4.4 ± 0.5 ^ab^		4.8 ± 0.6 ^a^	4.4 ± 0.5 ^ab^	
AVTCPK#8	1.8 ± 0.5 ^a^	2.2 ± 0.4 ^a^		1.8 ± 0.5 ^c^	2.2 ± 0.3 ^c^		2.4 ± 0.3 ^ab^	2.2 ± 0.4 ^b^	
AVTCPK#12	2.8 ± 0.2 ^a^	2.4 ± 0.4 ^a^		3 ± 0 ^abc^	2.4 ± 0.4 ^bc^		3 ± 0 ^ab^	2.4 ± 0.4 ^ab^	
AVTCPK#19	2.6 ± 0.4 ^a^	2.6 ± 0.3 ^a^		3.2 ± 0.5 ^abc^	2.6 ± 0.3 ^bc^		4.8 ± 1.2 ^a^	2.6 ± 0.3 ^ab^	
AVTCPK#24	2.2 ± 0.4 ^a^	1.8 ± 0.4 ^a^		2.4 ± 0.4 ^bc^	2.2 ± 0.5 ^c^		2.4 ± 0.4 ^ab^	2.2 ± 0.5 ^b^	
AVTCPK#25	2.4 ± 0.4 ^a^	2.2 ± 0.4 ^a^		2.6 ± 0.5 ^bc^	2.6 ± 0.3 ^bc^		2.6 ± 0.5 ^ab^	2.6 ± 0.3 ^ab^	
Mean	2.6	2.3		3.0	2.65		3.28	2.7	
	F-value	*p*-value	LSD	F-value	*p*-value	LSD	F-value	*p*-value	LSD
Genotype (G)	1.92	0.07	0.71	7.05	3.13 × 10^−6^	0.82	5.26	8.79 × 10^−5^	0.98
Treatment (T)	3.38	0.70	0.35	2.88	0.09	0.41	6.54	0.013	0.49
G*T	0.68	0.69	0.99	0.46	0.85	1.17	1.04	0.41	1.38

Same letters indicate no significance, while different letters indicate a significant effect. LSD indicates least significant difference. Values are presented as averages ± SEM.

**Table 4:** Chlorophyll content (SPAD value), carbon assimilation rate (A_sat_) (µmolm^−2^s^−1^), stomata conductance (gsw) (molm^−2^s^−1^), internal carbon concentration (Ci) (vpm), and internal water use efficiency (iWUE) (µmolmol^−1^) at 10 DAT.

Genotypes	Chlorophyll Content (SPAD Value)			Carbon Assimilation Rate (Asat)			Stomata Conductance (gsw)			Internal Carbon Concentration (Ci)			iWUE (A/gsw)		
	WW	WS		WW	WS		WW	WS		WW	WS		WW	WS	
AVTCPK#1	44.5 ± 1 ^bcd^	38.7 ± 2 ^de^		19.4 ± 0.6 ^a^	14.3 ± 0.6 ^ef^		0.265 ± 0.01 ^ab^	0.226 ± 0.01 ^abcdef^		319 ± 9.3 ^a^	288 ± 18.7 ^abc^		73.3 ± 2.4 ^defg^	63.4 ± 4.2 ^fg^	
AVTCPK#3	43.3 ± 1.3 ^bcd^	39.2 ± 1.2 ^cde^		17.2 ± 0.6 ^bc^	14.1 ± 0.2 ^f^		0.233 ± 0.02 ^abcde^	0.201 ± 0.01 ^cdef^		299 ± 3.8 ^ab^	279 ± 4.93 ^abcd^		75.2 ± 5.1 ^cdefg^	70.1 ± 2.54 ^defg^	
AVTCPK#6	51.0 ± 1.4 ^a^	46.6 ± 1.0 ^ab^		20.5 ± 0.3 ^a^	16.0 ± 0.3 ^cdef^		0.205 ± 0.01 ^bcdef^	0.172 ± 0.01 ^ef^		256 ± 15.2 ^bcd^	230 ± 12.3 ^d^		99.7 ± 1.3 ^a^	93.2 ± 3.24 ^abc^	
AVTCPK#8	44.3 ± 0.9 ^bcd^	41.5 ± 1.2 ^bcd^		19.1 ± 0.5 ^ab^	16.7 ± 0.3 ^cd^		0.268 ± 0.01 ^a^	0.235 ± 0.01 ^abcd^		307 ± 11.1 ^ab^	299 ± 13.8 ^ab^		72.3 ± 4.9 ^defg^	71.2 ± 1.8 ^defg^	
AVTCPK#12	40.8 ± 0.6 ^bcd^	33.7 ± 2.1 ^e^		16.3 ± 0.4 ^cde^	10.5 ± 0.4 ^g^		0.248 ± 0.02 ^abcd^	0.189 ± 0.03 ^def^		315 ± 11.1 ^a^	281 ± 3.7 ^abcd^		68.2 ± 6.1 ^efg^	58.4 ± 6.6 ^g^	
AVTCPK#19	50.9 ± 1.2 ^a^	45.4 ± 1.6 ^abc^		19.6 ± 0.4 ^a^	14.9 ± 0.4 ^def^		0.207 ± 0.01 ^abcdef^	0.169 ± 0.01 ^f^		268 ± 7.7 ^abcd^	237 ± 18.3 ^cd^		94.9 ± 1.4 ^ab^	88.0 ± 1.9 ^abcd^	
AVTCPK#24	46.7 ± 0.9 ^ab^	42.9 ± 0.8 ^bcd^		20.4 ± 0.6 ^a^	17.0 ± 0.3 ^bcd^		0.245 ± 0.02 ^abcd^	0.209 ± 0.01 ^abcdef^		323 ± 11.2 ^a^	310 ± 1.07 ^ab^		84.1 ± 3.1 ^abcde^	81.5 ± 2.1 ^abcdef^	
AVTCPK#25	45.1 ± 0.8 ^abc^	42.3 ± 0.9 ^bcd^		19.8 ± 0.4 ^a^	16.8 ± 0.26 ^cd^		0.262 ± 0.01 ^abc^	0.226 ± 0.01 ^abcdef^		323 ± 5.69 ^a^	319 ± 9.24 ^a^		76.7 ± 5.1 ^bcdefg^	74.5 ± 2.9 ^cdefg^	
Mean	45.80	41.26		19.04	15.01		0.24	0.21		301.45	280.42		80.52	75.03	
	F-value	*p*-value	LSD	F-value	*p*-value	LSD	F-value	*p*-value	LSD	F-value	*p*-value	LSD	F-value	*p*-value	LSD
Genotype (G)	18.15	4.74 × 10^−13^	3.92	33.08	<2 × 10^−16^	0.87	7.99	5.96 × 10^−7^	0.03	13.37	1.72 × 10^−10^	22.05	18.42	3.38 × 10^−13^	7.56
Treatment (T)	52.63	6.64 × 10^−10^	1.25	344.16	<2 × 10^−16^	0.43	38.07	5.17 × 10^−8^	0.01	14.51	0.0003	11.03	8.39	0.0051	3.78
G*T	0.73	0.63	6.31	3.5	0.00183	1.23	0.24	0.97	0.04	0.55	0.79	31.19	0.39	0.90	10.71

Same letters indicate no significance, while different letters indicate a significant effect. LSD indicates least significant difference. Values are presented as averages ± SEM.

**Table 5:** Chlorophyll content (SPAD value), carbon assimilation rate (Asat) (µmol m^−2^ s^−1^), stomata conductance (gsw) (mol m^−2^ s^−1^), internal carbon concentration (Ci) (vpm), and internal water use efficiency (iWUE) (µmol mol^−1^) at 20 DAT.

Genotypes	Chlorophyll Content (SPAD Value)			Carbon Assimilation Rate (Asat)			Stomata Conductance (gsw)			Internal Carbon Concentration (Ci)			iWUE		
	WW	WS		WW	WS		WW	WS		WW	WS		WW	WS	
AVTCPK#1	40.8 ± 1.8 ^abcdef^	30.3 ± 1.7 ^gh^		18.6 ± 0.4 ^abc^	10.3 ± 0.2 ^h^		0.227 ^abc^	0.147 ± 0.01 ^ef^		254 ± 5.5 ^abcde^	203 ± 3.4 ^ef^		81.8 ± 1.08 ^abc^	70.4 ± 2.1 ^cd^	
AVTCPK#3	39.3 ± 1.8 ^abcdefg^	31.9 ± 1.9 ^fgh^		17.0 ± 0.02 ^bcd^	12.8 ± 0.3 ^g^		0.217 ± 0.02 ^abcd^	0.149 ± 0.01 ^ef^		244 ± 7.2 ^bcde^	224 ± 7.7 ^cde^		82.5 ± 8.9 ^abc^	85.8 ± 1.9 ^abc^	
AVTCPK#6	48.0 ± 1.8 ^a^	38.4 ± 1.9 ^bcdefg^		19.7 ± 0.7 ^a^	9.79 ± 0.2 ^h^		0.185 ± 0.01 ^cde^	0.107 ± 0.01 ^fg^		237 ± 4.9 ^bcde^	210 ± 5.9 ^def^		106 ± 2.5 ^a^	91.9 ± 4.3 ^abc^	
AVTCPK#8	46.9 ± 0.6 ^abcd^	38.3 ± 1.2 ^cdefg^		20.2 ± 0.3 ^a^	15.2 ± 0.5 ^def^		0.255 ± 0.01 ^a^	0.177 ± 0.01 ^de^		301 ± 1.4 ^a^	256 ± 7.8 ^abcd^		80.3 ± 4.7 ^abc^	86.0 ± 2.7 ^abc^	
AVTCPK#12	37.5 ± 2.4 ^defg^	25.8 ± 1.8 ^h^		16.2 ± 0.3 ^cde^	4.34 ± 1.3 ^i^		0.221 ± 0.02 ^abc^	0.0863 ± 0.01 ^g^		231 ± 15.2 ^cde^	160 ± 5.1 ^f^		75.0 ± 5.9 ^bcd^	51.6 ± 14.6 ^d^	
AVTCPK#19	47.9 ± 0.6 ^ab^	37.1 ± 3.3 ^efg^		19.6 ± 0.2 ^a^	9.89 ± 0.5 ^h^		0.200 ± 0.01 ^bcd^	0.121 ± 0.01 ^fg^		247 ± 4.3 ^bcde^	210 ± 4.4 ^def^		97.7 ± 0.74 ^ab^	82.3 ± 5.3 ^a^	
AVTCPK#24	47.2 ± 0.5 ^abc^	38.2 ± 1.8 ^cdefg^		19.7 ± 0.2 ^a^	13.9 ± 0.4 ^efg^		0.24 ± 0.01 ^ab^	0.188 ± 0.01 ^cde^		305 ± 9.5 ^a^	285 ± 13.2 ^ab^		82.0 ± 1.5 ^abc^	73.8 ± 2.3 ^bcd^	
AVTCPK#25	42.8 ± 0.9 ^abcde^	33.9 ± 3.4 ^efgh^		18.7 ± 0.3 ^ab^	13.6 ± 0.5 ^fg^		0.248 ± 0.01 ^a^	0.188 ± 0.01 ^cde^		270 ± 11.0 ^abc^	257 ± 27.4 ^abcd^		75.7 ± 2.6 ^bcd^	72.5 ± 3.3 ^bcd^	
Mean	43.79	34.24		18.71	11.22		0.22	0.15		261.17	225.63		85.17	76.79	
	F-value	*p*-value	LSD	F-value	*p*-value	LSD	F-value	*p*-value	LSD	F-value	*p*-value	LSD	F-value	*p*-value	LSD
Genotype (G)	10.73	7.33 × 10^−9^	3.76	42.65	<2 × 10^−16^	0.96	21.95	8.446 × 10^−15^	0.02	20.28	4.62 × 10^−14^	20.63	8.37	3.12 × 10^−7^	10.56
Treatment (T)	102.51	6.46 × 10^−15^	1.89	974.23	<2 × 10^−16^	0.48	319.84	<2 × 10^−16^	0.01	47.37	2.99 × 10^−9^	10.32	10.06	0.002	5.28
G*T	0.28	0.96	5.33	17.25	1.32 × 10^−12^	1.36	4.03	0.0010	0.03	1.76	0.11	29.18	1.75	0.12	14.92

Same letters indicate no significance, while different letters indicate a significant effect. LSD indicates least significant difference. Values are presented as averages ± SEM.

**Table 6:** Carbon isotope discrimination (Δ^13^C) and aboveground biomass shown by eight *kabuli* chickpea genotypes.

Genotypes	Carbon Isotope Discrimination (Δ^13^C)			AGB (g Plant ^−1^)		
	WW	WS		WW	WS	
AVTCPK#1	24.5 ± 0.1 ^ab^	24.4 ± 0.03 ^ab^		10.5 ± 0.6 ^cd^	2.96 ± 0.3 ^f^	
AVTCPK#3	24.1 ± 0.2 ^ab^	23.9 ± 0.03 ^b^		5.00 ± 0.9 ^ef^	3.44 ± 0.7 ^f^	
AVTCPK#6	21.5 ± 0.3 ^de^	20.9 ± 0.3 ^e^		33.0 ± 2.1 ^a^	13.2 ± 0.2 ^c^	
AVTCPK#8	24.8 ± 0.2 ^a^	23.9 ± 0 ^b^		4.69 ± 0.67 ^ef^	4.39 ± 0.2 ^f^	
AVTCPK#12	24.5 ± 0.1 ^ab^	24.2 ± 0.2 ^ab^		4.25 ± 0.5 ^f^	2.45 ± 0.4 ^f^	
AVTCPK#19	22.4 ± 0.6 ^c^	22.1 ± 0.3 c^d^		26.9 ± 2.8 ^b^	9.38 ± 0.2 ^cde^	
AVTCPK#24	24.8 ± 0.2 ^a^	24.1 ± 0.4 ^ab^		6.58 ± 0.5 ^def^	3.64 ± 0.6 ^f^	
AVTCPK#25	24.8 ± 0.3 ^a^	24.0 ± 0.2 ^b^		5.43 ± 0.6 ^ef^	3.28 ± 0.4 ^f^	
Mean	23.9	23.4		12.06	5.38	
	F-value	*p*-value	LSD	F-value	*p*-value	LSD
Genotype (G)	47.51	2 × 10^−16^	0.51	115.5	<2 × 10^−16^	3.14
Treatment (T)	12.45	0.00078	0.25	179.3	<2 × 10^−16^	0.99
G*T	0.57	0.77	0.72	29.5	<2 × 10^−16^	5.05

Same letters indicate no significance, while different letters indicate a significant effect. LSD indicates least significant difference. Values are presented as averages ± SEM.

**Table 7:** Root length, root biomass, and root–shoot ratio shown by eight *kabuli* chickpea genotypes.

Genotypes	Root Length (cm)			Root Biomass (g plant^−1^)			R:S		
	WW		WS	WW		WS	WW		WS
AVTCPK#1	73 ± 3.75 ^abcd^		54.4 ± 10.3 ^cd^	3.69 ± 0.53 ^bc^		0.883 ± 0.07 ^d^	0.627 ± 0.07 ^ab^		0.489 ± 0.04 ^bc^
AVTCPK#3	58.8 ± 5.19 ^bcd^		58.8 ± 2.92 ^bcd^	1.54 ± 0.28 ^cd^		1.33 ± 0.30 ^cd^	0.528 ± 0.03 ^abc^		0.605 ± 0.04 ^abc^
AVTCPK#6	91.2 ± 1.32 ^a^		90.2 ± 1.6 ^a^	11.9 ± 1.3 ^a^		4.32 ± 0.27 ^b^	0.662 ± 0.05 ^ab^		0.462 ± 0.04 ^bc^
AVTCPK#8	66.8 ± 5.51 ^abcd^		65.5 ± 4.7 ^abcd^	1.61 ± 0.19 ^cd^		1.52 ± 0.12 ^cd^	0.633 ± 0.04 ^ab^		0.581 ± 0.04 ^abc^
AVTCPK#12	53.5 ± 8.34 ^cd^		51 ± 2.9 ^d^	1.08 ± 0.09 ^d^		0.661 ± 0.12 ^d^	0.468 ± 0.04 ^bc^		0.399 ± 0.04 ^c^
AVTCPK#19	84.4 ± 3.17 ^ab^		81.4 ±3.4 ^abc^	10.7 ± 1.23 ^a^		3.18 ± 0.22 ^bcd^	0.717 ± 0.04 ^a^		0.473 ± 0.04 ^bc^
AVTCPK#24	72.4 ± 9.83 ^abcd^		64.8 ± 2.1 ^abcd^	1.83 ± 0.24 ^bcd^		1.33 ± 0.17 ^cd^	0.508 ± 0.06 ^abc^		0.662 ± 0.03 ^ab^
AVTCPK#25	68.6 ± 4.12 ^abcd^		60.8 ± 8.1 ^bcd^	1.80 ± 0.13 ^bcd^		1.25 ± 0.17 ^cd^	0.586 ± 0.03 ^abc^		0.648 ± 0.03 ^ab^
Mean	70.92		66.01	4.28		1.81	0.59		0.54
	F-value	*p*-value	LSD	F-value	*p*-value	LSD	F-value	*p*-value	LSD
Genotype (G)	10.11	1.9 × 10^−8^	11.15	63.16	<2 × 10^−16^	1.00	3.82	0.0015	0.08
Treatment (T)	3.07	0.08	5.82	96.88	1.96 × 10^−14^	0.50	6.05	0.0015	0.04
G*T	0.67	0.69	15.76	21.25	1.70 × 10^−14^	1.46	5.72	0.0015	0.12

Same letters indicate no significance, while different letters indicate a significant effect. LSD indicates least significant difference. Values are presented as averages ± SEM.

**Table 8:** Eigenvectors, eigenvalues, and variance for the first two principal components for traits in eight genotypes of *kabuli* chickpea grown under water stress.

Parameters	PCA1		PCA2	
	Loading	R^2^	Loading	R^2^
SPAD10	0.20	0.04	0.20	0.04
SPAD20	0.15	0.02	0.27	0.08
A_sat_10	0.08	0.01	0.35	0.12
A_sat_20	−0.01	0.00	0.36	0.13
gsw10	−0.17	0.03	0.20	0.04
gsw20	−0.08	0.01	0.35	0.12
Ci10	−0.19	0.04	0.18	0.03
Ci20	−0.02	0.00	0.37	0.13
iWUE10	0.21	0.05	0.14	0.02
iWUE20	0.15	0.02	0.20	0.04
∆^13^C	−0.23	0.05	−0.02	0.00
SY	0.23	0.06	0.03	0.00
AGB	0.24	0.06	−0.03	0.00
HI	−0.17	0.03	0.26	0.07
Seed number	0.24	0.06	0.01	0.00
Pod weight	0.22	0.05	0.00	0.00
Pod number	0.24	0.06	0.02	0.00
PH	0.23	0.05	−0.07	0.01
PS	0.19	0.04	−0.06	0.00
Leaves	0.20	0.04	−0.06	0.00
DTF	0.23	0.05	−0.05	0.00
DTP	0.23	0.05	−0.04	0.00
DTM	0.23	0.05	0.01	0.00
Root length	0.23	0.05	0.06	0.00
Root weight	0.24	0.06	0.00	0.00
R:S	−0.07	0.01	0.34	0.11
WUE	0.19	0.04	0.20	0.04
Eigenvalue	17.72		7.14	
Variance explained, %	65.6		26.46	
Cumulative variance, %	65.6		92.10	

## Data Availability

The dataset underpinning the results of the study is available upon request from the corresponding author.
